# Bovine Milk-Derived Exosomes as a Drug Delivery Vehicle for miRNA-Based Therapy

**DOI:** 10.3390/ijms22031105

**Published:** 2021-01-22

**Authors:** Lorena del Pozo-Acebo, M-C López de las Hazas, Joao Tomé-Carneiro, Paula Gil-Cabrerizo, Rodrigo San-Cristobal, Rebeca Busto, Almudena García-Ruiz, Alberto Dávalos

**Affiliations:** 1 Laboratory of Epigenetics of Lipid Metabolism, Instituto Madrileño de Estudios Avanzados (IMDEA)-Alimentación, CEI UAM+CSIC, 28049 Madrid, Spain; lorena.delpozo@imdea.org (L.d.P.-A.); paulagilcab@gmail.com (P.G.-C.); almudena.garcia@imdea.org (A.G.-R.); 2 Laboratory of Functional Foods, Instituto Madrileño de Estudios Avanzados (IMDEA)-Alimentación, CEI UAM+CSIC, 28049 Madrid, Spain; joao.estevao@imdea.org; 3 Laboratory of Cardiometabolic Nutrition, Instituto Madrileño de Estudios Avanzados (IMDEA)-Alimentación, CEI UAM+CSIC, 28049 Madrid, Spain; rodrigo.sancristobal@imdea.org; 4 Department of Biochemistry-Research, Hospital Universitario Ramón y Cajal, IRYCIS, 28034 Madrid, Spain; rebeca.busto@hrc.es; 5 CIBER de Fisiopatología de la Obesidad y Nutrición (CIBEROBN), Instituto de Salud Carlos III (ISCIII), 28034 Madrid, Spain

**Keywords:** extracellular vesicles, miRNAs, exosomes, bovine milk, size exclusion chromatography

## Abstract

MicroRNAs (miRNAs) are small non-coding RNAs with a known role as mediators of gene expression in crucial biological processes, which converts them into high potential contenders in the ongoing search for effective therapeutic strategies. However, extracellular RNAs are unstable and rapidly degraded, reducing the possibility of successfully exerting a biological function in distant target cells. Strategies aimed at enhancing the therapeutic potential of miRNAs include the development of efficient, tissue-specific and nonimmunogenic delivery methods. Since miRNAs were discovered to be naturally transported within exosomes, a type of extracellular vesicle that confers protection against RNase degradation and increases miRNA stability have been proposed as ideal delivery vehicles for miRNA-based therapy. Although research in this field has grown rapidly in the last few years, a standard, reproducible and cost-effective protocol for exosome isolation and extracellular RNA delivery is lacking. We aimed to evaluate the use of milk-derived extracellular vesicles as vehicles for extracellular RNA drug delivery. With this purpose, exosomes were isolated from raw bovine milk, combining ultracentrifugation and size exclusion chromatography (SEC) methodology. Isolated exosomes were then loaded with exogenous hsa-miR148a-3p, a highly expressed miRNA in milk exosomes. The suitability of exosomes as delivery vehicles for extracellular RNAs was tested by evaluating the absorption of miR-148a-3p in hepatic (HepG2) and intestinal (Caco-2) cell lines. The potential exertion of a biological effect by miR-148a-3p was assessed by gene expression analysis, using microarrays. Results support that bovine milk is a cost-effective source of exosomes which can be used as nanocarriers of functional miRNAs with a potential use in RNA-based therapy. In addition, we show here that a combination of ultracentrifugation and SEC technics improve exosome enrichment, purity, and integrity for subsequent use.

## 1. Introduction

Exosomes are a subtype of extracellular vesicles (EVs) with a size range between 30 and 150 nm, which can be found in the extracellular spaces of tissues and in body fluids, including plasma, urine and milk [1]. Bioactive lipids, proteins, nucleic acids (such as DNA, mRNA and non-coding RNA), signaling molecules and receptors can be transported over distances within the protection of a lipid bilayer-enclosed structure such as exosomes [1]. Since exosomes can be isolated from body fluids, many studies are emerging with the aim of better understanding (1) how they are actively released from cells, (2) their involvement in cell-to-cell communication, and (3) their utility as biomarkers for early detection of disease through non-invasive liquid biopsy [2].

Although research in this field has grown rapidly in the last few years, a standard protocol for reproducible, cost-effective and exosome isolation has not been established yet. Originally, exosome isolation relied basically on ultracentrifugation-based techniques and, while these techniques remain the gold standard, other isolation methods have been developed, which make the most of the physicochemical and biochemical properties of exosomes (including size, solubility, density and immunoaffinity capture). However, each method has its own limitations and fails to isolate exclusively exosomes [3]. Several parameters must be considered when selecting the appropriate isolation method, including sample type, initial sample volume and downstream applications. Improved EV isolation methods may not only have an impact on the amount and purity of recovered EVs but may also result in the isolation of specific EV populations, with different sizes and functional characteristics and carrying certain RNA, protein and lipid profiles [4,5,6,7,8,9]. Thus, the challenge remains to develop mass-scalable methods to isolate EVs in a rapid, efficient, reproducible, cost-effective and clinically friendly manner.

Exosomes and their cargos do not exclusively originate from endogenous synthesis and may also be obtained from dietary sources. In the last decade, the number of studies reporting the isolation of EVs from foods (both from plant and animal origin) has increased substantially [10,11]. In particular, milk is a major source of EVs and their cargos may have a bioactive role in the consumer’s health [12,13,14,15,16].

Two different methods are commonly applied to isolate exosomes from milk: ultracentrifugation [17,18,19] and size-exclusion chromatography (SEC) [20,21]. However, ultracentrifugation has several drawbacks, such as the co-isolation of non-exosomal impurities [22], low reproducibility, potential damage of exosomes and low-throughput of samples [23]. Therefore, alternatives, like SEC, are being used more and more often [24,25]. Compared to ultracentrifugation, some studies report that EVs isolated by chromatographic methods have less contamination by non-vesicular proteins and macromolecule structures [26], better reproducibility and greater preservation of their biophysical and functional properties [9]. Other studies, however, argue that body fluids contain many nanoparticles (some non-vesicular) in the same size range as exosomes that can co-elute with them [27]. The main disadvantage of SEC could be the limited quantity of EVs recovered by unit of volume being required an additional supplementation method for exosome enrichment [3]. A combination of these methods may improve exosome enrichment, purity and integrity for subsequent therapeutic use.

In 1973, Plantz et al. showed the presence of extracellular vesicles in bovine skimmed milk [28], which is currently considered a potentially scalable source of exosomes [18]. Exosomes can serve as drug delivery vehicles for several reasons, which include the fact that they (i) present cross-species biocompatibility; (ii) have longer circulating half-life; (iii) are internalized by other cells; and (iv) carry a cargo of macromolecules from both hydrophilic and lipophilic source. In addition, exosomes can overcome both hematoencephalic [29] and placental barriers [30,31]. Exosome surface can also be engineered for targeted delivery and thus, tissue-specific biodistribution [32].

Among the several cargos transported within milk exosomes, non-coding RNAs—especially microRNAs (miRNAs), have found to be particularly abundant [33]. Interestingly, exosomes seem to increase miRNA stability by protecting them from RNase degradation [34]. miRNAs are small RNAs of 19 to 22 nucleotides in length that can regulate gene expression at the post-transcriptional level. More than 30% of human genes are predicted to be influenced by miRNAs, including genes involved in pathways of human diseases [35]. This fact, coupled to their intrinsic capacity to simultaneously modulate multiple targets, provides a mechanism to regulate a large suite of transcripts, offering a novel approach for therapeutic intervention. However, it is not clear whether miRNAs that are naturally transported in exosomes are able to exert genome regulation as some studies claim a minimum number of miRNA copies per cell is necessary for a biological effect to be exerted via RNAi or other molecular mechanisms [36]. Therefore, exosome-enrichment with the desired exogenous miRNA may be required to achieve biologically relevant effects on the gene expression of target cells.

Here, we aimed at developing an efficient and reproducible method for the isolation of bovine milk exosomes to be used as miRNA-delivery vehicles, based on a combination of ultracentrifugation and SEC. Purified bovine milk exosomes were loaded with a synthetic miRNA and their suitability as delivery vehicles was tested in hepatic (HepG2) and intestinal (Caco-2) human cell lines. Furthermore, the potential biological effect exerted by a miRNA loaded into bovine milk exosomes was assessed in both human cell lines by microarray analysis of differential gene expression profiles.

## 2. Results

### 2.1. Comparison between Isolation Methodologies of EVs from Bovine Milk

According to the recommendations from the International Society of Extracellular Vesicles (ISEV) at least three proteins, whose presence or absence in exosomes has been previously characterized, should be examined simultaneously to rule out the presence of cellular contamination in the preparations [37]. This could be done by including: one transmembrane or lipid-bound extracellular protein (CD9, CD63, CD81), one cytosolic protein (TSG101, Hsp70, Hsp90, Rab18, Rab7a, Annexin A1, A2, A7), and one intracellular protein absent (or under-represented) in exosomes but present in other types of EV (calnexin (endoplasmic reticulum), GM130 (Golgi), CYC1 (mitochondria)). Here, proteins Hsp90, CD63, TSG101 and calnexin were selected for evaluation. As caseins constitute the dominant protein in milk, representing about 80% of the total protein content [38], β-casein was evaluated as marker of sample purity.

#### 2.1.1. One vs. Two Ultracentrifugation Cycles

**Figure 1:**
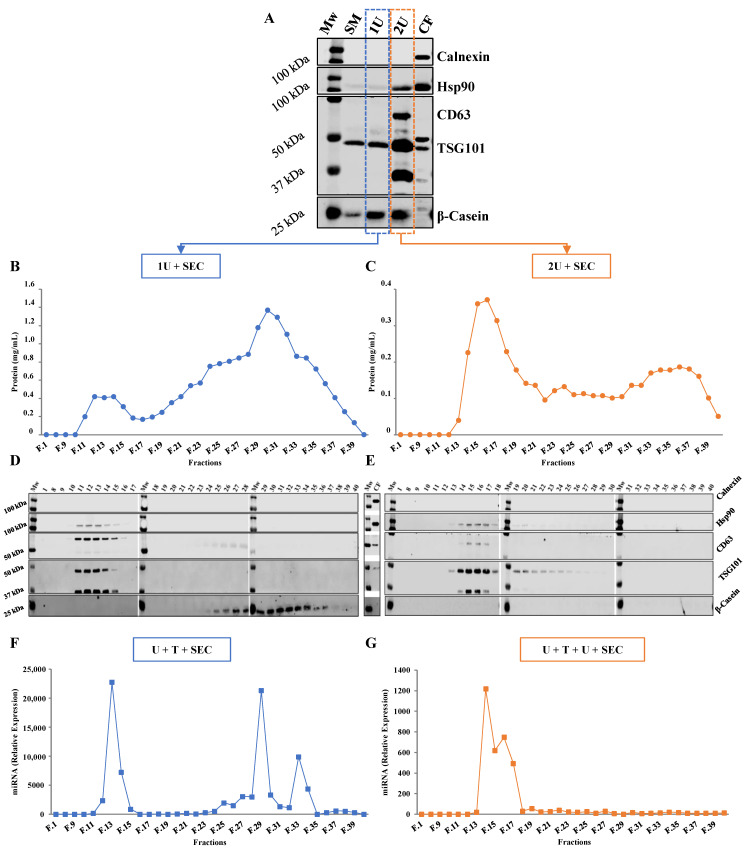
(**A**) Western Blot analysis of proteins present (Hsp90, CD63 and TSG101) or absent (calnexin) in exosomes and abundant in bovine milk (β-casein). Protein evaluation in: bovine skimmed milk (SM); exosomes obtained from SM by one (1U) or two (2U) ultracentrifugation steps; and the cellular fraction (CF). Equal amount of protein was loaded. Elution protein profile (F.1, F.8 to F.40) of bovine exosomes isolated by 1U followed by SEC (1U + SEC) (**B**) or 2U + SEC (**C**). Protein concentration (mg/mL) was estimated by the BCA assay. WB of SEC elution fractions from exosomes isolated by 1U + SEC (**D**) or 2U + SEC (**E**). Evaluation of Hsp90, CD63, TSG101, Calnexin and β-casein levels in each fraction (F.1, F.8 to F.40). Mimic hsa-miRNA-148a-3p elution profile (relative expression) of exosomes isolated from skimmed milk by U + T + SEC (**F**) or U + T + U + SEC (**G**). Mw: Molecular weight marker (Bio-Rad).

Exosomes obtained through one ultracentrifugation (1U) and two ultracentrifugations (2U) displayed the presence of TSG101 (±45 kDa), while Hsp90 (±90 kDa) and CD63 (±63 kDa) EV markers were only detected in 2U samples. Although the levels of Hsp90, CD63 and TSG101 increased were higher after the second ultracentrifugation step, contamination with casein proteins seems to persist since no reduction in β-casein was observed between 1U to 2U. These data suggest that even though the second ultracentrifugation step enriches the sample in exosomes, remains insufficient to remove all casein protein contamination. The absence of calnexin indicates there is a lack of cellular debris in skimmed milk and, consequently, in exosome samples (Figure 1A).

#### 2.1.2. Ultracentrifugation Followed by SEC

After the initial ultracentrifugation step(s) casein levels were still detected by Western Blot (Figure 1A). To determine whether SEC could further clean exosomes from contamination, this method was performed after the ultracentrifugation step(s) (named here as 1U + SEC or 2U + SEC). Forty sequential fractions of 500 µL were collected and the protein concentration of each fraction was determined. The protein elution profile of 1U + SEC (Figure 1B) shows two clear peaks (from fractions 11 to 16 and 21 to 37), whereas only one peak (14 to 19) is observed after 2U + SEC (Figure 1C). Then, fractions obtained after SEC were analyzed by WB to assess the relative content of EV markers and β-Casein (Figure 1D,E). EV markers (Hsp90, CD63 and TSG101) were detected in the fractions corresponding to the first peak observed after 1U + SEC (11 to 16), while β-casein was not, indicating that further EV purification was achieved. β-casein was identified in the fractions of the second peak, coinciding with the highest protein levels (25 to 37) (Figure 1D). The fractions of the first peak obtained from 2U + SEC (13 to 19) also displayed the presence of EV markers, especially TSG101. Furthermore, when equal volumes of samples were employed, β-casein was not detected in any fraction (Figure 1E), although protein signal did not disappear completely in the protein elution profile (Figure 1C). Finally, calnexin was not detected in any of the collected fractions, which indicates the absence of contamination by debris.

### 2.2. Loading Bovine Milk Exosomes with Exogenous RNAs

A synthetic miRNA, hsa-miR-148a-3p, with identical sequence and highly expressed in both human and bovine milk EVs was selected for exosome loading (Supplementary Figure S1). Exosomes isolated after the first ultracentrifugation step were chemically transfected with 100 µM of miRIDIAN hsa-miR-148a-3p. Loaded exosomes were then isolated by SEC (U + transfection (T) + SEC) or by an additional ultracentrifugation cycle followed by SEC (U + T + U + SEC). RNA was isolated from each fraction obtained in the miRNA elution profile and relative expression was calculated using PBS as a blank control (Figure 1F,G). Two peaks were observed in the miRNA elution profile of the U + T + SEC sample (Figure 1F), one between fractions 12 to 14 and the second one between fractions 29 to 35. The first peak of the mimic hsa-miR-148a-3p elution profile (Figure 1F) completely coincides with the first peak of the protein elution profile (Figure 1B), whose fractions are enriched in exosomes (Figure 1D). Moreover, the miRNA copies detected in the final fractions correspond with the highest protein level (29 to 32), and where casein was identified (Figure 1D). In the case of U + T + U + SEC (Figure 1G) only the first peak (14 to 17) was detected (Figure 1C), which also completely matches with the high exosome-enriched fractions (Figure 1E). These results confirm that exosomes have been loaded with mimic hsa-miR-148a-3p but the transfection efficiency may not have been total.

### 2.3. Purified Exosomes Loaded with a Synthetic miRNA Marginally Co-Elute with the Transfection Reagent

**Figure 2:**
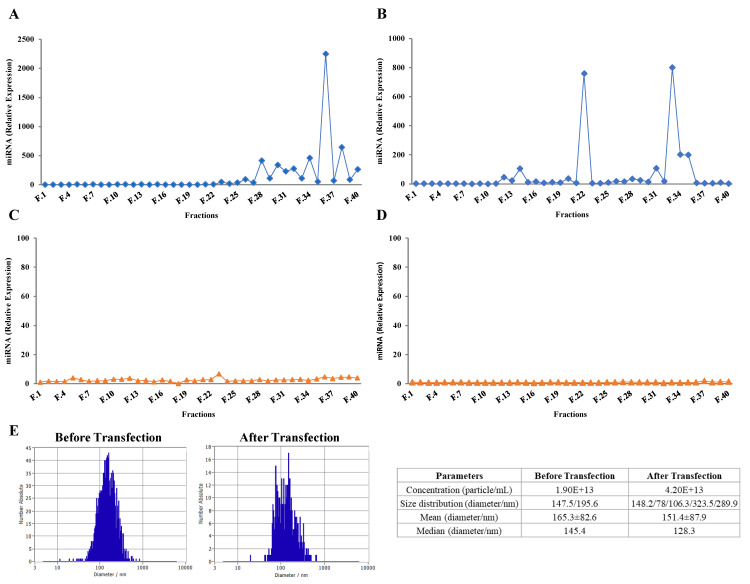
Mimic hsa-miR-148a-3p elution profile (RT-qPCR expression analysis). miRNA alone (**A**) or combined with lipofectamine after SEC (**B**). miRNA alone (**C**) and miRNA combined with lipofectamine after one ultracentrifugation step followed by SEC (**D**). Nanoparticle tracking analysis (NTA) before and after exosome transfection (**E**).

To discard the possibility that lipofectamine could be eluted complexed to exogenous RNAs and exosomes or to RNAs within the same size range of exosomes eluent, a comparison between the elution profile of either the miRNA alone or combined with lipofectamine was performed (Figure 2A,B, respectively). Two mimic hsa-miRNA-148a-3p solutions with or without lipofectamine were incubated at room temperature, for 15 min, then loaded onto a SEC column after which 40 sequential fractions were collected. The miRNA expression profile was calculated using PBS as control (Figure 2A,B). Several peaks were observed between fractions 28 to 39 of the miRNA without lipofectamine elution profile (Figure 2A), whereas one minor first peak (fractions 11 to 13) and a second one (fractions 33 to 35) were observed in the miRNA plus lipofectamine elution profile (Figure 2B).

Moreover, when comparing the miRNA elution profile of transfected exosomes (Figure 1F) with the one of free miRNA plus lipofectamine (Figure 2B), the fractions of the first peak coincide, suggesting that lipofectamine could be co-eluting with exosome-enriched fractions in samples separated by U + T + SEC. However, performing an additional ultracentrifugation cycle after the transfection step (U + T + U + SEC) seems to remove any free miRNA copies, which had not been incorporated inside exosomes (Figure 2C), together with any remaining traces of lipofectamine (Figure 2D). These data suggest that lipofectamine has a minimal effect on the miRNA elution profile, mainly resulting in the elution of some miRNA copies in the initial fractions (coinciding with exosome-enriched fractions).

To determine if size and concentration of exosomes are affected by miRNA transfection, nanoparticle tracking analysis (NTA) was assessed in selected samples before and after transfection (Figure 2E). No major changes in size distribution and diameter were observed, suggesting that miRNA transfection minimally influence major physical characteristics of exosomes.

### 2.4. Exogenous RNAs Are Incorporated into Exosomes

**Figure 3:**
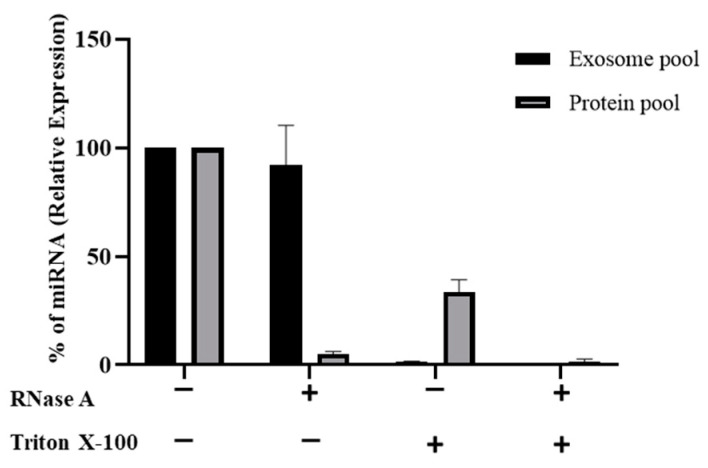
RNase protection assay. The survival percentage of mimic miR-148a-3p was quantified by RT-qPCR in exosome and protein pooled samples treated with RNase A, Triton X-100, or both. Results are expressed in percentage of protected miRNA compared to the respective control.

A RNase A protection assay was performed to confirm that miRNA was confined inside exosomes instead of being adhered to the exterior exosomal membrane. Thus, first peak fractions (corresponding to the highest levels of mimic hsa-miR-148a-3p) where EV markers were detected (here named as the exosome pool), and second peak fractions, coinciding with miRNA traces and with the highest levels of protein (named as the protein pool). The transfected exosome/protein pools were treated with either RNase A or Triton X-100 (to disrupt vesicles lipid bilayer) alone, or with the combination of both. Total RNA was isolated and RT-qPCR was performed. The percentage of protected miRNA was calculated by comparing each group with its corresponding control (non-treated transfected exosome/protein pools) (Figure 3). Results showed no differences in miRNA degradation between RNase A-treated and non-treated exosomes, whereas miRNA levels were barely detected in RNase A-treated protein samples. Moreover, miRNA levels could not be detected when both pools were treated with RNase A plus Triton X-100, evidencing the protective effect of the exosome lipid bilayer against RNase A activity and the effective incorporation of miRNA into exosomes [39,40,41]. Curiously, miRNA was neither detected in Triton X-100- treated exosomes.

### 2.5. miRNAs Transported within Exosomes Are Taken up by Mammalian Cells In-Vitro

**Figure 4:**
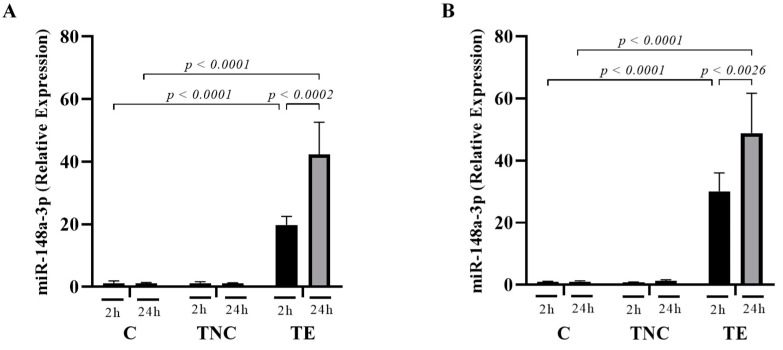
Cellular uptake of mimic hsa-miR-148a-3p. Relative expression of hsa-miR-148a-3p in HepG2 (**A**) and Caco-2 (**B**) cells exposed to either 100 µg protein/mL of exosomes transfected with 100 µM of mimic hsa-miR-148a-3p (TE) or negative control miRNA (TNC). Non-treated cells were used as controls (C). Values were standardized with RNU6. Values are means ± SEMs; *n* ≥ 3 independent experiments. *p*-value < 0.05 was considered as statistically significant.

Fractions from the first peak collected by U + T + SEC, in which the presence of EV markers and the absence of protein contaminants had been confirmed, were pooled to evaluate the potential of exosomes as miRNA-delivery vehicles. HepG2 (Figure 4A) and Caco-2 (Figure 4B) cells were treated with 100 µg protein/mL of miRNA mimic negative control (TNC) or mimic hsa-miR-148a-3p (TE), for 2 or 24 h. Non-treated cells were used as controls (C). Five hundred ng of total RNA were used for RT-qPCR. In HepG2 cells, a significant 20- and 45-fold increase in the concentration of miR-148a-3p was observed at 2 h and 24 h, respectively (Figure 4A), while, in Caco-2, levels were raised by 30- and 48-fold, respectively (Figure 4B). No accumulation of miR-148a-3p was observed in both control groups (C and TNC). This data demonstrates that mimic hsa-miR-148a-3p-enriched exosomes were successfully internalized by cells in a time-dependent way.

### 2.6. miRNAs Delivered by Bovine Milk-Derived Exosomes Exert Gene Expression Modulation

**Figure 5:**
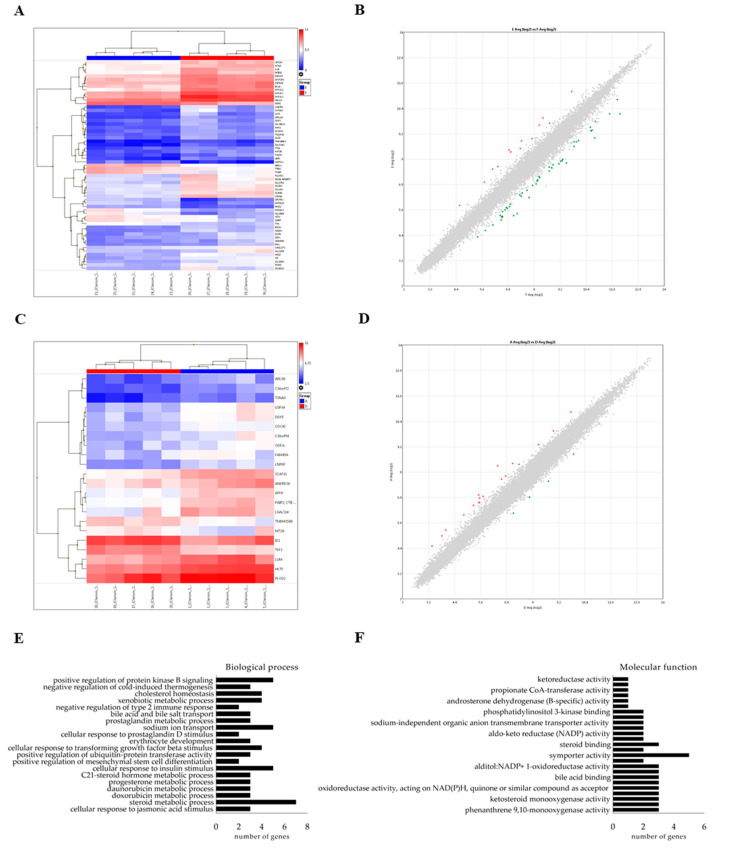
(**A**) Hierarchical clustering of HepG2 gene profiles after exposure to hsa-miR-148a-3p (TE) or mimic negative control miRNA (TNC), during 24 h. (**B**) Scatter plot of differentially expressed genes in HepG2 cells. (**C**) Hierarchical clustering of Caco-2 gene profiles after exposure to hsa-miR-148a-3p or mimic negative control miRNA, during 24 h. (**D**) Scatter plot of differentially expressed genes in Caco-2 cells. (**E**) In-silico analysis of the possible biological processes occurring after the exposure to hsa-miR-148a-3p. (**F**) In-silico analysis of the possible molecular functions involved in the response to hsa-miR-148a-3p exposure.

A transcriptomic analysis using microarrays was performed to assess the possible biological effects resulting from hsa-miR-148a-3p delivery to human cells via bovine-milk exosomes. HepG2 and Caco-2 gene profiles were compared after exposure to TNC or TE, for 24h. The heat map representation of each analysis showed appropriate clustering between TNC and TE groups in both cell lines (Figure 5A,C). A scatter plot of differentially expressed genes is depicted in Figure 5B,D.

Fifty-six differentially expressed genes were identified in HepG2 (13 up-regulated and 43 down-regulated) and twenty-one in Caco-2 cells (18 up-regulated and 3 down-regulated). The full list of modulated genes is shown in Supplementary Tables S1 and S2.

Functional analysis of differently expressed genes was performed using the Genecodis4.0 database. This analysis displayed several neural system-related processes, among others (Supplementary Tables S1 and S2). In hsa-miR-148a-3p-treated cells, the GO biological processes identified were related to steroid metabolic processes, cellular response to insulin response, sodium ion transport, positive regulation of protein kinase B signaling, cellular response to transforming growth factor beta stimulus, xenobiotic metabolic processes or cholesterol homeostasis, among others (Figure 5E). Molecular function analysis suggested symporter activity, phenanthrene 9,10-monooxygenase activity, trans-1,2-dihydrobenzene-1,2-diol dehydrogenase activity, ketosteroid monooxygenase activity or steroid dehydrogenase activity, among others (Figure 5F). KEGG pathway analysis revealed an association with steroid hormone biosynthesis (hsa00140) (FDR < 0.0315894).

## 3. Discussion

One of the major limitations for the use of exosomes in a clinical setting, with diagnosis and therapeutic purposes, is the lack of standardized isolation methods and characterization techniques. There is a need for more efficient, specific, reliable, and reproducible EV extraction methods so that all downstream applications in this field can be normalized and reproduced.

Several studies support the notion that diet-derived exosomes and/or their cargos may exert bioactive actions in human health. Indeed, Manca et al., reported that bovine milk exosomes administered through oral delivery reach different tissues (intestinal mucosa, spleen, liver, heart or brain) [42]. Here, we provide a small-scalable exosome isolation method, based on a combination of ultracentrifugation and size-exclusion chromatography, using bovine milk (one of the richest sources of EVs) as the sample source. Moreover, recent studies have shown that miRNAs are particularly abundant cargos in milk exosomes, emerging as bioactive components potentially responsible for beneficial actions on consumers’ health [13,15,43]. However, it is still a subject of controversy whether diet-derived exosomal miRNAs are sufficient to achieve biologically relevant effects on gene expression in target cells [44]; but, it seems that plant ncRNAs could be good bioactive candidates for miRNA therapy [45]. Some studies have estimated that the required intracellular levels for target gene regulation to occur must be greater than 100 copies [46] or between 1000 and 10,000 copies per cell [47], although success will also depend on the target subcellular location [46] and on the number of target transcripts [48]. Therefore, it seems reasonable that enriching exosomes with selected exogenous miRNAs is important to enhance the possibilities of achieving a measurable activity. In this work, we enriched bovine milk isolated exosomes with hsa-miR-148a-3p to study their potential suitability as drug delivery agents for miRNA-based therapy.

Despite the fact that differential ultracentrifugation is the most widely used isolation method, contaminants, including protein aggregates, can be co-precipitated with exosomes [22]. To address this problem, we first determined if caseins were removed after one ultracentrifugation or two ultracentrifugation cycles (Figure 1A). Results indicated that, even though a second ultracentrifugation step enriches the sample in exosomes (levels of Hsp90, CD63 and TSG101 were increased compared with 1U), full removal of protein contamination is not achieved. These data confirm that ultracentrifugation allows for the isolation of bovine milk EVs, but it must be combined with additional isolation techniques to accomplish enhanced exosome purity [3,22].

According to the literature, SEC could be a complementary method to eliminate non-exosomal impurities co-isolated with EVs during ultracentrifugation [21,25]. Therefore, we tested the efficiency of 1U + SEC compared with 2U + SEC in isolating bovine-milk exosomes. 1U + SEC elution protein profile (Figure 1B) presented a higher second protein peak compared with 2U + SEC, indicating that a second ultracentrifugation step decreases total protein contamination. Indeed, 1U + SEC (Figure 1D,E) revealed that exosome fractions (11 to 16 and 13 to 19, respectively) lacked β-casein. However, the elution profile of 1U + SEC (Figure 1B) showed high protein concentration in the intermediate fractions (16 to 24) in which neither EV markers nor β-casein were found. Some remaining protein contaminants were also observed in 2U + SEC fractions (Figure 1C), although β-casein was not detected. Despite the fact that α-casein is the most abundant protein in bovine milk [38], its elution profile is quite similar to β-casein, thus the absence of β-casein in SEC fractions also indicates an absence of α-casein. These results are in concordance with Blans et al., who showed that the first peak was free of all types of casein proteins [20]. These data suggest that caseins and other major proteins, which precipitate with EVs after one and two ultracentrifugation cycles (as observed in Figure 1A), can only be removed by SEC.

Mammalian milk is an abundant source of miRNAs. While a large number of miRNAs have been identified in milk exosomes, several studies indicate that enrichment is only found in a few cases, such as miR-148a-3p (Supplementary Tables S3 and S4). This miRNA is highly expressed in human and bovine milk exosomes (Supplementary Figure S1) and is involved in the regulation of genes associated with different cellular processes [49,50,51,52,53]. Several methods can be used to incorporate molecules to EVs; here, lipofectamine transfection was employed. Comparing the miRNA elution profiles of U + T + SEC and U + T + U + SEC, one initial peak within fractions 11 to 16 (Figure 1F) and 14 to 17 (Figure 1G), respectively, completely matches with the first peak of each protein elution profile (Figure 1B,C), and these fractions are enriched in exosomes (Figure 1D,E). The second peak detected in Figure 1F could be due to the remaining copies of hsa-miR-148a-3p, which were not incorporated into exosomes and might be found in the free form or forming miRNA-casein protein complexes. This second peak was not observed in Figure 1G, suggesting that a second ultracentrifugation step after miRNA incorporation removes free miRNA copies. These results confirm that, even though the transfection efficiency was not flawless, exosomes were transfected with mimic hsa-miR-148a-3p. However, it is important to consider the influence that RNA sequence and secondary structure of miRNAs could have in their incorporation efficiency. Indeed, we do not discard that other miRNAs may have different behavior [54].

As previously mentioned, for therapeutic use, exosomes need to be highly purified to avoid immunological responses due to other reagents. There is one remote possibility that the transfection reagent, a cationic lipid particle, could elute complexed to exogenous RNAs together with exosomes or with RNAs within the same size of exosomes eluent. As observed in Figure 2A,B, lipofectamine may affect the miRNA elution profile to some extent. Lipofectamine reagent contains cationic lipid subunits that can form liposomes in an aqueous environment. The cationic liposomes can bind to negatively charged nucleic acid molecules, overcoming the electrostatic repulsion of lipidic membranes. The RNA-containing liposomes can fuse with the exosome lipid membrane allowing them to cross into EVs. Therefore, mimic-hsa-miR-148a-3p complex with lipofectamine leading to the early elution of miRNA copies that were not incorporated to exosomes (fractions 12 to 14) (Figure 2B). Comparing the miRNA elution profile of transfected exosomes (Figure 1F) with the one of miRNA plus lipofectamine (Figure 2B), we can observe that the first peak fractions coincide, suggesting that lipofectamine could be co-eluting with exosome-enriched fractions. However, in Figure 2D, the hsa-miR-148a-3p could not be detected, denoting that the additional ultracentrifugation step introduced after miRNA transfection can remove the remaining lipofectamine molecules.

Overall, results suggest that the introduction of a second ultracentrifugation step after the miRNA transfection step can eliminate any remaining free miRNA copies (those that were not internalized in exosomes) and lipofectamine traces. However, exosome isolation methods based solely in ultracentrifugation are not sufficient to remove all protein contaminants. In addition, ultracentrifugation potentially alters the physical and functional properties of exosomes [23]. Consequently, according to the literature and considering the correlated drawbacks, one ultracentrifugation cycle can be considered as a suitable small-scalable primary isolation method, but it should be combined with another separation technique. In this study, SEC proved to be an effective approach for exosome purification. Furthermore, SEC preserves the membrane integrity and biological activity of exosomes [9]. Therefore, exosomes isolated by one ultracentrifugation, loaded with exogenous miRNA, and further purified by SEC were used for downstream analysis.

Since RNAs and other molecules may remain attached to the outside part of the vesicle membrane instead of being internalized by exosomes, their incorporation into the vesicle was tested here. According to the bibliography, exosomes can prevent RNases from degrading miRNAs present inside [34]. The RNase A protection assay performed seemed to discard the possibility of miRNAs being bound to the exterior vesicle membranes (Figure 3). However, the second peak pool treated with RNase A showed near complete miRNA degradation, confirming that some miRNA copies were not internalized during the transfection procedure (Figure 3). Our data confirm that RNAs enclosed in exosomes are protected from RNase activity [39,40,41]. Nevertheless, it is important to note that miRNA degradation was also observed in both pools treated exclusively with Triton X-100, suggesting that adding this detergent at 1% is not a good approach to test miRNA incorporation into lipidic membrane vesicles.

In-vitro uptake analysis of exogenous hsa-miR-148a-3p (loaded into bovine milk exosomes) revealed statistically significant concentration increases in both HepG2 and Caco-2 cell lines. Moreover, the increment found between 2 and 24 h of exosome exposure suggests that the absorption of bovine milk-derived exosomes is time dependent in these cells. These results corroborate several studies stating that exposure time is an important variable to consider in these type of studies [55]. By contrast, no differences were observed between C and TNC groups, indicating that the absorption of bta-miR-148a, which is naturally transported within bovine milk exosomes, did not influence endogenous miRNA levels, at least at the concentrations tested here, and, therefore, dietary miRNAs may not have a measurable (relevant) impact on the gene expression of target cells [56].

Exosomes loaded with a mimic negative control miRNA were used as controls to assure that target-cell gene expression modulation was specifically caused by the exogenous hsa-miR-148a-3p (loaded into exosomes) and not due to an unspecific response to potentially bioactive exosome cargos (such as lipids and proteins) or to an increase in the amount of ribonucleic acid molecules. Several genes were modulated by hsa-miR-148a-3p and the ones showing higher repression were *AKR1C1*, *AKR1C2*, *CYP3A5*, *CAB39L*, *ODAM* and *NEGR1*, among other for HepG2 cells and *TFF3*, *TMEM150B* and *ID1* for Caco-2 cell line.

Other studies showed that miR-148a-3p target genes were involved in pathways associated with proliferation [51,57] lipid metabolism [53] and adipogenesis [50], among other processes [49,52]. Here, common to these studies, an association with lipid metabolism pathways was identified in the GO analysis. It is likely that the previously undescribed modulated genes in response to miR-148a-3p found here are particular to the model cell types used. Indeed, to the best of our knowledge, this was the first transcriptomic analysis aimed at assessing the effect of the overexpression of miR-148a-3p, using exosomes as the delivery vehicle, performed in HepG2 and Caco-2 cell lines.

Delivery of miRNA-enriched exosomes resulted in highly efficient overexpression of the candidate miRNA in recipient mammalian cells in-vitro. Furthermore, we confirmed that exosome-delivered extracellular miRNA is functional in recipient cells since host gene expression was modulated. Collectively, we developed a protocol for the isolation and use of bovine milk exosomes as successful delivery vehicles for bioactive extracellular miRNAs, with a potential use in RNA-based therapy.

Lipid nanoparticles has been previously used to deliver miRNAs and other drugs to cells [58]. For example, a cationic lipid 1,2-dioleoyl-3-trimethylammonium-propane solid lipid nanoparticles (SLN) (average size from 48 to 141 nm) or a dimethyldioctadecylammonium bromide were synthesized (average size 200 nm) and loaded with miR-200c or miR-34a, respectively [58,59]. After 24h incubation, miRNAs were taken up by cells in higher amounts than those of lipofectamine complexes [58] and SLNs showed better protection of miRNA from degradation than that of lipofectamine [59]. In this context, bovine milk exosomes also show a good protection of those miRNAs incorporated within the vesicles as exemplified here, suggesting their suitability for miRNA delivery as those previously shown for lipid nanoparticles.

## 4. Materials and Methods

### 4.1. Preparation and Purification of Milk Exosomes

**Figure 6:**
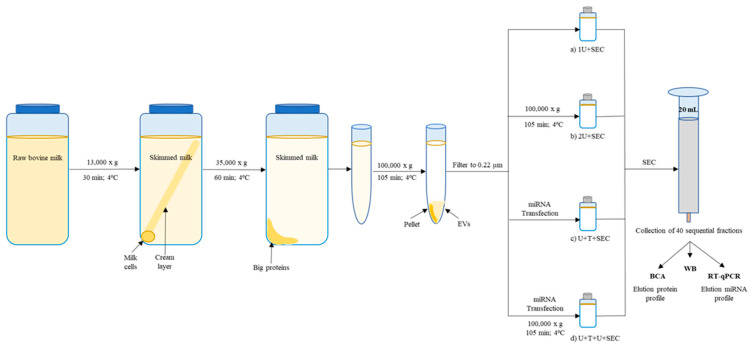
Summary of the EV isolation approaches. Raw bovine milk was skimmed by differential centrifugation to remove cells, fat globules and large proteins. Ultracentrifugation of 25 mL of skimmed milk (SM) results in a solid pellet containing most of the SM casein, a supernatant of milk serum, and a viscous phospholipid rich soluble concentrate positioned adjacent to the casein pellet containing EVs (called fluffy layer). EV fluffy layers were filtered through 0.22 µm syringe filters. Afterwards, 700 µL of filtered EVs were enriched through two different approaches: (**a**) SEC (1U + SEC) or (**b**) additional ultracentrifugation followed by SEC (2U + SEC). After first ultracentrifugation, EVs loaded with exogenous miRNA by the transfection method were then isolated by (**c**) SEC (U + T + SEC) or (**d**) additional ultracentrifugation followed by SEC (U + T + U + SEC). Forty sequential fractions of 500 µL were collected and their miRNA and protein elution profiles were subsequently obtained by carrying out different methodology (BCA, Western Blot and/or RT-qPCR analysis).

Bovine milk was directly collected in a local farm (Madrid, Spain) and stored at 4 °C until use [60]. Sequential centrifugation and ultracentrifugation steps were performed to isolate the exosomal fraction. Briefly, milk was centrifuged at 13,000× *g* in an Avanti Centrifuge J-26XPI (Beckman Coulter, Brea, CA, USA), using a JLA 16.250 rotor, for 30 min, at 4 °C to eliminate cells and milk fat globules. The supernatant was then centrifuged at 35,000× *g*, for 60 min, at 4 °C to remove large proteins, such as casein, and cell debris. The supernatant (skimmed milk) was then ultracentrifuged in an OPTIMA L-90K Ultracentrifuge (Beckman Coulter), using a 50.2 Ti rotor, at 100,000× *g* in 25 mL bottles, for 105 min, to precipitate EVs. The resultant supernatant was discarded. Additional ultracentrifugation steps and/or SEC were performed to enrich and purify exosome samples (Figure 6).

#### 4.1.1. Exosomes Isolated through Two Ultracentrifugations

The EV fluffy layer was resuspended in 25 mL of phosphate-buffered saline (PBS) and a second ultracentrifugation step at 100,000× *g*, for 105 min, was carried out. The exosome-enriched pellet was resuspended in 700 µL of PBS until a homogenous suspension was obtained and, subsequently, filtered through 0.22 µm syringe filters (Millex^®^-GP, Merck Millipore, Burlington, MA, USA) to eliminate large size particles. Samples were stored at −80 °C until use.

#### 4.1.2. Exosomes Isolated through One or Two Ultracentrifugation Cycles Followed by SEC

After the first or second ultracentrifugation step, the fluffy layer was resuspended in PBS until a homogenous suspension was obtained. EV pellet was, subsequently, filtered through 0.22 µm syringe filters (Merck Millipore). Filtered exosomes (700 µL) were loaded onto a SEC column for EV purification, as previously described [61] with some modifications. Briefly, 20 mL of Sepharose CL-2B (CL2B300, Sigma Aldrich, Madrid, Spain) were stacked in a 20 mL syringe (BD Plastik, Madrid, Spain) (final matrix length: 12 cm and diameter: 1.6 cm) and equilibrated with 0.22 µm filtered PBS (pH 7.4). According to the manufacturer’s instructions, this setting will collect particles with a size distribution between 60 and 200 µm in separated fractions. Elution was performed by gravity using 0.22 µm filtered PBS (pH 7.4) and 40 sequential fractions of 500 µL were collected.

### 4.2. Protein Determination

Protein concentration of each fraction was determined by the BCA method (Thermo Scientific, Waltham, MA, USA), using bovine serum albumin (BSA) as standard, and following the manufacturer’s instructions.

### 4.3. Western Blot

To determine the presence of exosomes, 20 µL of each fraction or 50 µg/protein of each sample were used for protein detection. Protein samples lysed in reducing conditions were separated on 10% sodium dodecyl polyacrylamide/bisacrylamide gels, transferred onto nitrocellulose membranes (0.22 µm Millipore) and blocked with 2.5% skimmed milk (Bio-Rad, Hercules, CA, USA), for 30 min, at room temperature. Membranes were then incubated with appropriate primary antibodies: anti-Hsp90 (610418, BD, Madrid, Spain), anti-CD63 (bs-1523R, BIOSS, Woburn, MA, USA), anti-TSG101 (A303-506A, Bethyl, Montgomery, TX, USA), anti-calnexin (A303-694A, Bethyl) or anti-β-casein (ab112595, abcam, Cambridge, UK) at 4 °C (overnight). After incubation, membranes were exposed to secondary anti-rabbit or anti-mouse conjugated antibodies, with either Alexa FluorTM 680 or IRDye^®^ 800, for 45 min at room temperature. Blots were washed three times with TBST buffer after each incubation step and visualized using an Odyssey^®^ infrared imaging system (LI-COR, Lincoln, NE, USA). Image Studio Lite 5.2.5 software was employed for image processing.

### 4.4. miRNA Loading and Nanoparticle Tracking Analysis

miRIDIAN hsa-miR-148a-3p mimic (synthetized by Dharmacon^®^) was used for the transfection of isolated bovine milk exosomes. A solution (100 µL) of 100 µM miRIDIAN hsa-miR-148a-3p mimic diluted in siRNA 1× Buffer (Thermo Fisher) was mixed with 30 µL of Lipofectamine 2000 reagent (Invitrogen, Thermo Scientific) and incubated, for 15 min, at room temperature. 700 µL of filtered exosome solution was added to the mixture. After 30 min of additional incubation at room temperature, the mixture was kept at 4 °C to minimize Lipofectamine action until further purification.

To determine the size and distribution of exosomes before and after miRNA loading, a nanoparticle tracking analysis was performed. Equivalent samples before and after transfection were diluted in PBS and exosome concentration and size were obtained using the ZetaView NTA system (Particle Metrix, Germany).

### 4.5. RNase A Treatment and RNA Isolation

To confirm that mimic hsa-miR-148a-3p incorporation was correct, 500 µL of transfected exosomes were incubated for 30 min at 37ºC with or without 10 µg/mL of RNase A (Ribonuclease A R6513-10MG, Sigma Aldrich) or with a mixture of 10 µg/mL RNase A and 1% of TRITON 100-X (Sigma Aldrich) [39]. Total RNA was subsequently purified using QIAzol Lysis Reagent (Qiagen, Madrid, Spain) by the chloroform/phenol method [62].

### 4.6. RT-PCR and qPCR

cDNA synthesis was performed with 500 ng of RNA using miScript II RT Kit (Qiagen), according to the manufacturer’s instructions. The cDNA product was then submitted to qPCR, in a 7900HT Fast Real-time PCR system (Applied Biosystems) with a 384 well plate format, using miScript SYBR Green PCR kit (Qiagen) and hsa-miR-148-3p specific oligo (Isogen LifeSciences, Utrecht, The Netherland) at 95 °C for 10 min, followed by 40 cycles at 95 °C for 15 sec and 58 °C for 1 min. Reactions were run in duplicate, miRNA expression was normalized with cel-miR-39-3p spike-in (Qiagen) or RNU6 cell housekeeping gene. Relative quantification was calculated by the 2^−ΔΔCt^ method.

### 4.7. Treatment of Mammalian Cells with Bovine Milk Exosomes

Human liver cancer cells (HepG2) and colorectal adenocarcinoma cells (Caco-2), obtained from the American Type Culture Collection (ATCC, USA), were cultured in Dulbecco’s Modified Eagle’s Medium (DMEM) with high glucose (Lonza), supplemented with 10% fetal bovine serum (FBS) (Sigma Aldrich), 1% L-glutamine (Gibco, Thermo Scientific) and 1% antibiotics (100 mg/mL penicillin and streptomycin) (Gibco). Cells were cultured at 37 °C in a 5% CO_2_ humidified incubator. Culture medium was replaced every two days. PBS was used to wash the cells.

HepG2 and Caco-2 cell lines were seeded in 6- and 12-well plates (Corning Costar^®^, Sigma Aldrich) at an initial density of 385,000 and 250,000 cells/well, respectively. Cells were incubated for approximately 24 h until 70% of confluency was reached. Subsequently, cells were washed and then cultured in DMEM supplemented with 0.5% FBS, 1% L-glutamine and 1% antibiotics, containing 100 µg/mL of EV-protein measured by BCA. Cells were incubated with previously isolated exosomes, which were first transfected with 100 µM of mimic hsa-miR-148a-3p (TE) or mimic negative control (TNC), during 2 or 24 h. Cells with basal culture media (CE) were used as controls. Each treatment was performed in quadruplicate. For each time point, the supernatant was discarded, cells were washed twice, collected in QIAzol Lysis Reagent (Qiagen) and stored at 80 °C until RNA extraction. 500 ng of RNA of each sample were used in RT-qPCR.

### 4.8. Microarray Analysis

Gene expression profiles for HepG2 and Caco-2 were performed using the 4 × 44 K complete human genome platform from Affymetrix (Clariom S Assays). Five biological replicates per group were included. Each microarray contains approximately 22,900 unique human genes and transcripts. Briefly, cDNA was synthesized from total RNA using One-cycle cDNA Synthesis Kit (Affymetrix, Santa Clara, CA, USA). cDNA was fragmented and hybridized to the Affymetrix matrix following the manufacturer’s instructions. Finally, after washing, cartridges were scanned in a GeneChip^®^ 3000 scanner for fluorescence signal acquisition. Data was normalized using the RMA method. Genes with a FDR lower than 0.1 were considered as statistically significant. Differential gene expression was assessed with the Bioconductor Limma package.

### 4.9. Pathway Analysis

Pathway analysis of miR-148a-3p-modulated genes was performed using Genecodis4.0 database [63,64,65]. For functional analysis, only gene ontology (GO) terms found to be over-represented (adjusted *p*-value < 0.05) in the gene input list after false discovery rate correction (following the hypergeometric statistical test) were included.

### 4.10. Statistical Analysis

Results are expressed as mean ± standard error of the mean. Data normality was tested using the Kolmogorov-Smirnov test. Parametric methods were used for analytical statistics. Non-parametric data were log transformed prior to statistical analyses. t-tests were performed to establish statistical significances between control and experimental groups. Differences were considered significant when the *p*-value was below 0.05. Version 7 of the GraphPad Prism program was used to perform the statistical analyses.

## 5. Conclusions

Our finding suggests that bovine milk represents a promising cost-effective source of exosomes, which can be used as nanocarriers of functional miRNAs for RNA-based therapy. Indeed, exosome-transported miR-148a-3p can be delivered and taken up by cells, in-vitro, and exert a biological effect through the modulation of gene expression. Moreover, a combination of ultracentrifugation and SEC improves exosome enrichment, purity and integrity for subsequent use.

## Data Availability

The data presented in this study are available in the article and supplementary material.

## Supplementary Materials

Supplementary materials can be found at https://www.mdpi.com/1422-0067/22/3/1105/s1. Supplementary Figure S1: Selection of major miRNAs from milk.; Supplementary Table S1: Modulated genes in HepG2 cell line in response to exosomes loaded with mimic hsa-miR-148a-3p.; Supplementary Table S2: Modulated genes in Caco-2 cell line in response to exosomes loaded with mimic hsa-miR-148a-3p.; Supplementary Table S3: Most abundant and common miRNAs of human breast milk exosomes from five previously published studies.; Supplementary Table S4: Most abundant and common miRNAs of bovine milk exosomes from five previously published studies.
